# Bacterial Concentration and Diversity within Repetitive Aliquots Collected from Replicate Continuous-Flow Bioreactor Cultures

**DOI:** 10.2174/1874285800802010060

**Published:** 2008-05-23

**Authors:** Tawni L Crippen, Cynthia L Sheffield, Kathleen Andrews, Roy Bongaerts, David J Nisbet

**Affiliations:** 1 Southern Plains Agricultural Research Center, Agricultural Research Service, U.S. Department of Agriculture, College Station, Texas, USA; 2 Flow Cytometry Laboratory, Institute of Food Research, Norwich Research Park, Colney, Norwich, UK

**Keywords:** Bioreactor, cecal, chicken, continuous-flow culture, repetitive sampling.

## Abstract

The aim of this study was to determine the reproducibility of small volume repeat sampling from replicate bioreactors with stabilized continuous-flow chicken cecal bacterial communities. Bacterial concentration and diversity were analyzed by phenotypic, biochemical and ribotype analysis. Significant differences in concentrations and variations in diversity were found in replicate bioreactors.

## INTRODUCTION

In the current climate of increased restrictions on the use of antibiotics to control pathogenic bacteria, a thorough understanding of the microbial ecology of the gastrointestinal tract is important. Insight into the role of the commensal microbial communities in preventing establishment of detrimental organisms in the gastrointestinal tract is required to develop prophylactic strategies that maintain the health of commercial flocks of chickens. Modulation of this environment by pre- and probiotics has demonstrated promising results. The bioreactor offers a model for the study of gastrointestinal bacterial communities and for the production of probiotic formulations.

The ability to grow bacteria under steady-state conditions for extended periods by supplying a continuous renewal of nutrients and replacement of media makes continuous-flow bioreactors a valuable tool for research and for commercial producers of probiotic materials. The resulting nearly constant environment serves as a platform allowing the production of bacterial cultures requiring sampling in a reproducible manner for batch consistency [1-3]. In previous studies, culture efficacy as a probiotic was ascertained by routine removal of a 1 ml planktonic aliquot from the continuous flow culture vessel [1, 4-6]. The efficacy testing protocol was replicated by repeatedly utilizing culture fluid from the same bioreactor vessel, indicating the expectation that each of the aliquots removed were identical. The assumption that any aliquot taken from a thoroughly mixed combination of cecal contents will result in an identical sample of equal bacterial concentration and diversity has not been explored. Studies measuring the frequency of components of mixed samples, such as pollen taxa from honey samples, have demonstrated that it is invalid to assume all populations within a mixed sample are equally represented in every aliquot sampled [7, 8].

Replicate bioreactor cultures from the same thoroughly mixed sample of avian cecal material were established. First, repetitive sampling of the planktonic component, in volumes consistent with inoculum preparations were analyzed to determine if all aliquots consisted of like bacterial profiles within the same bioreactor. Second, replicate bioreactors, initiated from aliquots of the same mixed cecal material, as a model of gastrointestinal colonization resulting from the inoculation, were characterized. We found consistency within subsets of the bacterial populations in repetitive sampling of the bioreactor planktonic component, but some variability in the specific species composition of the resulting bacterial community within bioreactors initiated from one source of mixed cecal material.

## MATERIALS AND METHODOLOGY

### Birds

German Lohmann Selected Leghorn (LSL) layer chicks were obtained on day of hatch from Hy-Line International, Inc. (Bryan, TX). The birds were housed in floor pens on pine shavings and provided with electrical heat lamps. Birds were provided water and a balanced, unmedicated, corn-soybean based feed ration *ad libitum* for 7 days (Poultry Science Department, Texas A&M University, College Station, TX). The feed ration met or exceeded nutrient levels recommended by the National Research Council [9].

### Bioreactor and Sampling Design

The ceca were aseptically harvested in two sets (A and B) from 150 chicks and the cecal contents from each set of birds were combined and thoroughly mixed under sterile, anaerobic conditions as previously described [10]. Three (I, II and III) replicate bioreactors (Bioflo^®^ 110 Fermentor/Bioreactor, New Brunswick Scientific Co, Inc., Edison, NJ) were initiated from the thoroughly mixed cecal contents of set A and three (IV, V and IV) bioreactors from set B. For each bioreactor, an aliquot of 1.5 g of cecal material was combined with 9 ml of modified Viande Levure medium, consisting of 10g L^-1^ tryptose, 5g L^-1^ yeast extract, 5g L^-1^ sodium chloride, 2.4g L^-1^ beef extract, 0.6g L^-1^ cysteine hydrochloride, and 2.5g L^-1^ dextrose(Fisher Scientific International Inc., Hampton, NH), and added into the 0.5 L of media in each bioreactor as previously described [1]. 

Cultures were kept under continuous-flow conditions at a flow rate of 0.8 ml/min and flushed with a constant a stream of O_2_-free CO_2_ to maintain anaerobiosis. The bioreactor was monitored for pH and adjusted to maintain a stable pH 6.2 ± 0.3 for the first 48 hr. Cultures were then allowed to equilibrate undisturbed for 3 weeks. After the equilibration period, 1 ml aliquots were collected for pH measurement and bacterial quantification, isolation and characterization. The bioreactor volume turnover time was 24 h to approximate the evacuation rate of the chicken cecum (once/day); samples were taken no more than once daily to avoid any dilution affect [11]. The planktonic component was sampled 11 times over a three week period. Culture pH was also monitored. 

### Bacterial Isolation and Preliminary Identification

Bioreactor culture material was sampled and subsets of bacterial populations were quantified by growth of a 10μl aliquot on selective media in triplicate as previously described [12]. Briefly, trypticase soy agar plates with 5% sheep blood (TS-blood agar; BVA Scientific, San Antonio, TX) was used for the recovery and enumeration of total aerobic microbial species and for the determination of hemolytic reactions, which are important differentiating characteristics for bacteria, especially *Streptococcus* species. MacConkey agar (Becton Dickinson, Sparks, MD) was used to select gram-negative bacteria and enumerate lactose fermenting coliforms, such as *Escherichia coli* (*E. coli)*, and *Klebsiella spp* [13]. Chromogenic *E. coli/Coliform* agar (CHROMagar, Paris, France) was used as a confirmatory test for**presumptive* E. coli* isolated from MacConkey agar. Rogosa agar (Becton Dickinson, Sparks, MD) was used for selective cultivation of chemoorganotrophic bacteria requiring nutritionally complex media containing fermentable carbohydrates, such as *Lactobacillus spp. *and* Pediococcus *spp. mEnterococcus agar (Becton Dickinson , Sparks, MD) was used for the enumeration of *Enterococcus* spp. Nonselective enumeration of obligate and facultative anaerobes was performed using *Brucella* agar with 5% horse blood (*Brucella*-blood agar; Anaerobe Systems, Morgan Hill, CA). Bacteroides bile esculin agar (BBE; Anaerobe Systems, Morgan Hill, CA) was used to isolate organisms from the *Bacteroides fragilis* group, and Veillonella agar (Becton Dickinson, Sparks, MD) was used to selectively isolate *Veillonella* spp.

Aerobic bacteria were isolated and preliminarily identified by streaking 10 µl aliquots of the continuous-flow mixed planktonic culture component onto TS-blood agar, Brilliant Green Agar (BGA; Becton Dickinson, Sparks, MD), CHROMagar *E. coli* and Orientation, MacConkey, mEnterococcus, and Rogosa plates. The plates were then incubated at 37°C for 24 h. Isolates were subcultured onto fresh media to assure cultural purity. Individual aerobic isolates were identified using ribotype analysis and Analytical Profile Index (API, bioMerieux, Inc., Durham, NC) matching [13-16].

Anaerobic bacteria were isolated and preliminarily identified by streaking aliquots onto *Brucella*-blood agar, Phenylethyl alcohol agar (Becton Dickinson, Sparks, MD), Veillonella, and BBE plates. Plates were then incubated anaerobically for 48-72 h at 37°C. Isolates were subcultured onto fresh media to assure cultural purity. All anaerobic isolates were tested for aerotolerance by incubation at 37°C for 24-48 h in a standard 5% CO_2_ incubator. Subcultured, purified isolates were plated onto TS-blood agar and incubated for 24 h at 37°C (facultative anaerobes) or *Brucella*-blood agar (obligate anaerobes) and incubated anaerobically at 37°C for 48-72 h, for subsequent definitive identification using automated ribotyping and API matching.

### Enumeration

Total aerobic bacterial population, lactose fermenting coliforms (coliforms), *Enterococcus spp.* and total anaerobic population levels were enumerated by serial dilution plating onto TS-blood agar, MacConkey, mEnterococcus agars or *Brucella*-blood agar plates, respectively. Results are reported as colony forming units (cfu) /ml.

### Ribotype Characterization

A RiboPrinter^®^ Microbial Characterization System (RMCS; DuPont Qualicon, Inc. Wilmington, DE) was used for all ribotype analyses. Isolates from the bacterial lawn were collected following an overnight culture analysed following manufacturers instructions using lytic enzymes (DuPont Qualicon, Inc.). Briefly, DNA was cleaved using the restriction endonuclease *Eco*RI and fragments separated by gel electrophoresis and analysed using a modified southern hybridization blotting technique. The DNA was hybridized with a labeled rRNA operon prob derived from *Escherichia coli*, and the bands were detected by chemiluminescence. The resulting image was captured and transferred to the RMCS database and data were normalized to a standard marker set (DuPont Qualicon, Inc.).

The resulting riboprint patterns were compared to the 6448 *Eco*RI riboprint patterns in the DuPont database and a 900 *Eco*RI riboprint pattern custom in-house database (USDA, ARS, College Station, TX). Characterisation of the bacteria consisted of combining profiles with a similarity ≥ 93%, as calculated by the proprietary algorithm of the RMCS, to form a dynamic ribogroup that reflected the genetic relatedness of the isolates. Ribotype identification similarities of less than 85% was confirmed using, API Staph, API 20E, API 20NE, API 20 Strep, and Rapid ID 32A (bioMerieux, Inc.) manual identification test strips.

### Data Analysis

Three replicate bioreactors were established for each set of combined cecal material. Eleven samples per bioreactor were analyzed for bacterial enumeration and characterization; once after three weeks of equilibration and followed by ten replicate samples over the subsequent three weeks. Descriptive statistics were generated and presented in table formats. The geometric mean was calculated as the n-th root of the product of n numbers (Microsoft Office Excel 2003, Microsoft Corp.) Data were analyzed using commercially available statistical software (JMP version 5.0.1, SAS Institute Inc., Cary, NC) [17]. Comparison of differences in cfu/ml among replicate bioreactor samples were analyzed by non-parametric Kruskall-Wallis test followed by a ChiSquare comparison (*P*0.05 for Table **1** and *P*0.01 for Table **2**).

## RESULTS AND DISCUSSION

Probiotics are supplements containing potentially beneficial microorganisms which, when administered in adequate composition and quantity, confer a health benefit to the host. In the poultry industry, one target health benefit includes competitive exclusion leading to the extinction of invading pathogenic competitors. To produce a probiotic, the collection and bioreactor culturing of bacteria from the gastrointestinal tract is done to establish a replica, including rare individuals, mimicking a beneficial and protective commensal bacterial community. Subsequent sampling from the bioreactors is done to prepare inocula for identical composition of the culture for various purposes. However, the probability of collecting one or more organisms can be affected by sample size and the frequency of the organism within the environment being sampled.

First, we should note enumeration of bacteria on non-selective versus selective media often yields counts that at first do not seem compatible. However, bacterial enumeration on non-specific media is affected by several factors including fitness of each competing species, initial number of each species, and individual species growth rates. Selective media by definition eliminates much of the competition for nutrients and resources by selecting for a limited number of species. Thus, bacteria that flourish on selective media may be less productive when faced with competition from several other species as would be found in a non-selective media culture, therefore growth rates and concentrations of individual bacteria species diminish on non-selective media [18]. This leads to a situation where the enumeration of a specific species or genus is considerably higher on selective media than its contribution to a "total" count on non-selective media. As a result, the total bacterial counts are not additive for each of the selective counts, as seen for the *Enterococcus* and coliform counts characterized in this study (Table **1**).

The bioreactor culture material was sampled and subsets of bacterial populations quantified by growth on selective and non-selective media. At three weeks in culture the concentration of *Enterococcus* spp., coliforms, aerobic, and anaerobic bacteria from each culture was determined and the presence of bacterial populations quantified in serial samples taken over a three week period (Table **1**). We found that repetitive 1 ml sampling from the same bioreactor over a three week period resulted in samples which consistently represented the source planktonic component in numbers of anaerobic, aerobic, coliform and *Enterococcus* subsets of bacteria. The mean of the ten repetitive samples did not differ significantly (*P*0.05) from the initial three week counts in any culture.

However there were differences (*P*(0.01) between the replicate bioreactors initiated with the same source material (Table **2**). Within set A, the sample population numbers measured in all subsets, coliforms, *Enterococcus*, aerobic and anaerobic bacterial populations, were significantly different between bioreactors. There was high variation in individual *Enterococcus* counts from bioreactor A-III. Measurement of the anaerobic bacterial populations, on *Brucella*-blood agar, was slightly, but significantly different in bioreactor A-III and measurement of coliforms in bioreactor A-II was slightly lower. Within set B samples, the quantity of coliforms and *Enterococcus* bacterial populations differed slightly, but significantly between bioreactors.

Spatial distribution of organisms affects their probability of collection. Aggregation causes a violation of the assumption required by the binomial theory that organisms are identically and independently distributed. Venette *et al*. [19] demonstrated that aggregation of commodity units during sampling leads to incorrect estimates of frequency and renders confidence limits unreliably small [19]. Therefore, in order to maintain a desired probability of collection, the sampling requirements must increase as the degree of unit aggregation increases. Many bacterial species are known to aggregate [20-28]. In addition, it is thought that adherent bacteria are relevant to the protection conferred by competitive exclusion and therefore are an expected component of a probiotic culture [29]. *Enterococcus faecalis*, for example, expresses plasmid encoded surface proteins, which act as aggregation substances that mediate adhesion between bacteria during conjugation and is a common species found adhering to surfaces [30, 31]. Thus, the diversity within each aliquot extracted from a complex system could vary due to aggregating species. The end result being inconsistencies in the final population cultured from each aliquot. Aggregation can also affect the enumeration of bacteria and may account for some of the variation we reported in bacterial quantification. Consideration should be given to sample size when using aliquots for inoculation from cultures with known aggregating species.

The characterization of bacteria isolated from the respective bioreactors, identified isolates from six genera (Table **3**). In set A, bioreactors II and III had identical planktonic composition and bioreactor I contained 6 of the 7 bacterial populations found in the other two cultures. In set B, bioreactors IV and V had identical planktonic composition and bioreactor VI contained 5 of the 6 bacterial populations found in the other two cultures. While *Clostridium* was prevalent in the planktonic component of set A, it was detected in only one planktonic component from set B. *E. coli* was detected throughout the set B bioreactors, but was not found in bioreactor A-I. Three species, *Enterococcus faecalis*, *Klebsiella pneumoniae* and *Pediococcus acidilactici* were detected in every bioreactor. The pH of the bioreactors within set A cultures ranged from 4.0 to 5.9; within set B cultures from pH 4.1 to 5.7.

The very nature of the richly diverse microbial constituents of the gastrointestinal tract makes consistent sampling problematic. Regardless of the sampling approach, when dealing with bacteria, the reconstitution of the gastrointestinal environment is not only limited by the difficulty of comprehensively harvesting all species, but by our present technological inability to artificially culture all bacterial species. Therefore, the collection any of individual organism for *in vitro* studies almost certainly results in a misrepresentation of its true frequency in the *in vivo* population. We found inconsistencies in the bacterial composition of bioreactors initiated from the same source material. Certain species were consistently represented, while other species were more variable in representation. These species likely represent a minor species within the community or a species less amenable to the artificial culture environment. Even when minor species are evenly distributed within the sample, the aliquot size may influence uptake and isolation of these more scarce species. When using the sample as an inoculate, the sample size is limited by the quantity of culture material within the vessel and by the size of the animal being inoculated. While we found variability in the analysis of the bacterial community initiated from the same source material, it should be noted that positives from selective media demonstrate sampling outcomes in which the presence of the individual is detected. However, the detection of a minor species is problematic since sampling does not provide sufficient evidence to prove that a species is absent. If the number of individual bacteria is below a threshold level it may not be sampled with a significant degree of confidence.

The effects of pooling fecal samples for the detection of pathogenic bacteria demonstrated a lower efficiency of detection where the prevalence of the target bacteria was diluted [32, 33]. Dilutions of cecal competitive exclusion material tested for their ability to protect chicks demonstrated that aliquots of 10^-8^ g of material did not retain sufficient quantity and/or diversity to be efficacious [34]. Stavric *et al*. [29] revealed a progressive loss of efficacy with dilution of washed ceca, whole ceca and feces, indicating that bacteria relevant to protection were not present at higher dilutions of the material, implying that a threshold quantity of specific bacterial species may be required for the establishment of the proper mix of protective community members.Adjustments in population density, diversity, and adhesion which occur during the artificial culturing period likely play a part in this observation.

In conclusion, the aim of this study was to determine the reproducibility of small volume repeat sampling from a bioreactor. We analyzed bacterial concentration and diversity within stabilized continuous-flow chicken cecal bacterial communities initiated by replicate aliquots taken from thoroughly mixed samples. Commercialization of probiotic cultures developed in continuous-flow bioreactors requires the reproducibility of bacterial efficacy within aliquots. Replicate bioreactors were established from samples of pooled cecal material from layer chicks. After a steady state was reached, the planktonic components were sampled repetitively over three weeks and then the planktonic component was characterized by phenotypic, biochemical and ribotype analysis. There were no significant differences in bacterial concentrations of repetitive samples from the same bioreactor. However, there were significant differences in bacterial concentrations and variations in bacterial diversity in replicate bioreactors initiated from the same stock material.

## Figures and Tables

**Table 1.** Geometric Mean (cfu/ml) of Subsets of Bacterial Populations of Each Replicate Bioreactor Culture

Set	Subset:	Coliforms		Enterococcus		Aerobic		Anaerobic	
	Bioreactor	3 week^a^	10-rep^b^	3 week	10-rep	3 week	10-rep	3 week	10-rep
A	I	3.77 x10^5^	7.73 x10^6^	1.59 x10^7^	2.94 x10^7^	1.53 x10^7^	2.62 x10^7^	2.02 x10^7^	2.82 x10^7^
	II	1.27 x10^5^	1.49 x10^5^	1.50 x10^7^	2.24 x10^7^	2.96 x10^7^	5.39 x10^7^	1.20 x10^7^	1.54 x10^7^
	III	7.49 x10^5^	7.01 x10^6^	6.08 x10^7^	1.01 x10^9^	1.86 x10^7^	3.06 x10^7^	6.20 x10^7^	1.06 x10^8^
B	IV	1.02 x10^5^	2.06 x10^5^	3.18 x10^7^	5.79 x10^7^	3.04 x10^7^	5.54 x10^7^	3.90 x10^7^	7.77 x10^7^
	V	6.50 x10^5^	3.55 x10^6^	3.00 x10^7^	6.14 x10^8^	5.88 x10^7^	7.49 x10^7^	5.60 x10^7^	9.11 x10^7^
	VI	1.39 x10^5^	1.61 x10^5^	1.22 x10^8^	2.30 x10^8^	3.69 x10^7^	5.98 x10^7^	3.90 x10^7^	7.80 x10^7^

^a^ = samples taken after 3 weeks of bioreactor equilibration.

^b^ = ten replicate samples taken over a 3 week period post equilibration.

**Table 2.** Comparison of Median (cfu/ml) and 95% Confidence Intervals (CI) of Subsets of Bacterial Populations from Repeat Sampling of Each Replicate Bioreactor Culture

	Subset:	Coliforms			Enterococcus			Aerobic			Anaerobic		
Set	Bioreactor	upper 95%Cl	lower 95%Cl	median^‡^	upper 95%Cl	lower 95%Cl	median	upper 95%Cl	lower 95%Cl	median	upper 95%Cl	lower 95%Cl	median
A	I	2.92 x10^7^	3.22 x10^6^	7.13 x10^6 ^^a^	3.25 x10^7^	2.69 x10^7^	3.17 x10^7^ ^a^	3.12 x10^7^	2.27 x10^7^	3.05 x10^7^ ^a^	7.74 x10^7^	7.82 x10^6^	3.27 x10^7^ ^a^
	II	6.11 x10^5^	2.45 x10^4^	1.48 x10^5^ ^b^	3.10 x10^7^	1.93 x10^7^	2.93 x10^7^ ^a^	5.76 x10^7^	5.06 x10^7^	5.57 x10^7^ ^b^	2.53 x10^7^	1.03 x10^6^	1.64 x10^7^ ^a^
	III	2.22 x10^7^	3.36 x10^4^	7.19 x10^6^ ^a^	9.55 x10^10^	-2.74 x10^10^	1.13 x10^8^ ^b^	3.75 x10^7^	2.63 x10^7^	3.51 x10^7^ ^a^	1.18 x10^8^	9.55 x10^7^	1.64 x10^7^ ^b^
B	IV	2.00 x10^6^	1.46 x10^5^	1.90 x10^5^ ^c^	6.64 x10^7^	5.16 x10^7^	6.35 x10^7^ ^c^	6.27 x10^7^	4.97 x10^7^	6.05 x10^7^ ^c^	7.80 x10^8^	7.75 x10^7^	7.78 x10^7^ ^c^
	V	1.36 x10^7^	2.09 x10^6^	3.30 x10^6^ ^d^	5.07 x10^11^	-1.82 x10^11^	5.46 x10^7^ ^c,d^	1.77 x10^8^	3.62 x10^7^	9.74 x10^7^ ^c^	1.13 x10^8^	7.74 x10^7^	1.07 x10^7^ ^c^
	VI	3.46 x10^5^	7.07 x10^7^	1.78 x10^5^ ^d,c^	2.47 x10^8^	2.16 x10^8^	2.43 x10^8^ ^d^	1.39 x10^8^	1.96 x10^7^	5.79 x10^7^ ^c^	7.80 x10^7^	7.80 x10^7^	7.80 x10^7^ ^c^

^‡^ n=10.

^a,b^= groups not connected by the same letter are significantly different within Set A (p < 0.01). Each bacterial subset considered independently.

^c,d^= groups not connected by the same letter are significantly different within Set B (p < 0.01). Each bacterial subset considered independently.

**Table 3.** Characterization of Bacterial Species Diversity within each Bioreactor Culture

	Set A			Set B			Identification Match	
Bacterial species	I^1^	II	III	IV	V	VI		
Clostridium spp	+	+	+	-	-	+	94%	API^2^
Esherichia coli	-	+	+	+	+	+	92%	Ribotype^3^
Enterococcus faecalis	+	+	+	+	+	+	95%	Ribotype
Enterococcus faecium	+	+	+	+	+	+	91%	Ribotype
Klebsiella pneumoniae	+	+	+	+	+	+	92%	Ribotype
Pediococcus acidilactici	+	+	+	+	+	+	94%	Ribotype
Proteus mirabilis	+	+	+	+	+	-	85%	Ribotype
pH^4^	5.9	5.8	4	4.1	4.9	5.7		
	± 0.1	± 0.1	± 0.0	± 0.0	± 0.1	± 0.0		

^1^Roman numeral designates one of three replicates bioreactor cultures, within Set A or B.

^2^Rapid ID 32A, Analytical Profile Index, bioMerieux, Inc.

^3^RiboPrinter Microbial Characterization System, DuPont Qualicon, Inc.

^4^Mean pH ± standard error of culture during replicate sampling (n = 11).
